# Atherogenic Activation of Human Vascular Smooth Muscle Cells by Monosodium Urate Crystals

**DOI:** 10.3390/gucdd1030016

**Published:** 2023-08-14

**Authors:** Ru Liu-Bryan, Tracy Guo, Jennifer Lee, Robert Terkeltaub

**Affiliations:** 1 VA San Diego Healthcare System, San Diego, CA 92161, USA; 2 Division of Rheumatology, Department of Internal Medicine, Seoul St. Mary’s Hospital, College of Medicine, Catholic University of Korea, Seoul, Republic of Korea

**Keywords:** hyperuricemia, gout, monosodium urate crystals, artery, vascular smooth muscle cells, IL-6, eicosanoids, atherosclerosis

## Abstract

Gout is strongly associated with atherosclerosis and other cardiovascular comorbidities. Furthermore, sites of extra-articular monosodium urate (MSU) crystal deposits in gout can include heart valves and atherosclerotic artery plaques, but with unclear effects therein. Hence, we seminally explored cultured vascular smooth muscle cell (VSMC) responsiveness to MSU crystals. To limit confounding effects, we cultured human aortic VSMCs under serum-free conditions to assess MSU crystal effects on VSMC differentiation and function, differentially expressed genes (DEGs) via RNA sequencing, and selected atherogenic changes in cytokines and the lipidome. MSU crystals induced p38 phosphorylation, IL-6, and VSMC vacuolization with dysregulated autophagy. MSU-crystal-induced DEGs included decreased late-stage autophagosome maturation mediator GABARAPL1, decreased physiologic VSMC differentiation regulators (LMOD1 and SYNPO2), increased ATF4, CHOP, and the intrinsic apoptosis signaling pathway in response to ER stress, and neointimal atherogenic nuclear receptors (NR4A1 and NR4A3). MSU crystals alone increased the levels of cholesterol biosynthetic intermediates 14-demethyl-lanosterol (14-DML), desmosterol, and zymosterol. Adding MSU crystals increased oxidized LDL’s capacity to increase intracellular 27-OH cholesterol, and MSU crystals and oxidized LDL synergistically induced a marked release of arachidonate. In conclusion, MSU crystals deposited in arterial media and neointima have the potential to dysregulate VSMC differentiation and proteostasis, and to induce further atherogenic effects, which include enhanced VSMC loading of oxidized cholesterol intermediates and release of IL-6 and arachidonic acid (AA).

## 1. Introduction

Gout is strongly associated with atherosclerosis and other cardiovascular comorbidities [1,2]. Furthermore, MSU crystals have been reported to deposit in a broad range of extra-articular sites in gout, which include certain cardiac and arterial tissues [3], and in the renal medulla in patients with relatively severe forms of tophaceous gout [4]. Also, case studies of patients with chronic, tophaceous disease have reported MSU crystal deposits in cardiac valves, the myocardium, and the cardiac conducting system [3,5–9]. Moreover, small, tophus-like intimal and adventitial coronary artery lesions have also been reported [10]. Notably, inflammatory macrophages express xanthine oxidase (XO) [11], and XO has been reported to be significantly upregulated in the macrophage-rich atherosclerotic plaques of patients with symptomatic central nervous system (CNS) ischemia, compared to plaques of those without CNS symptoms [12,13]. XO and urate are particularly enriched in the shoulder and subendothelial regions of such carotid atherosclerotic plaques, and increased XO and urate aggregate in plaque [13].

Using a highly sensitive micro-optical coherence tomography approach augmented by cross-polarized light microscopy and uricase treatment, MSU crystals have been detected in the coronary atherosclerotic plaques of gout patients [14,15]. The number of MSU crystals counted significantly correlated with histology-derived intimal thickening and was significantly greater in gout patients compared with non-gout patients [15]. Nonetheless, the robust deposition of basic calcium phosphate (BCP) and cholesterol crystals in atherosclerotic plaques has limited the interpretation of studies using less refined means to evaluate MSU crystal deposits in arterial lesions, such as imaging whole arteries by dual-energy computed tomography (DECT) and performing gross arterial histology [3,10,16–19].

BCP crystal deposition is intimately involved in the inflammatory process in atherogenesis and also plaque repair processes, so much so that the volume of calcification is a standard biomarker of plaque burden [20]. Cholesterol crystals activate the complement and the NLRP3 inflammasome and stimulate the plaque inflammatory process [21]. However, the effects of MSU crystals on atherogenesis and activation of arterial cells other than mononuclear phagocytes remain unclear. This contrasts with the well-recognized role of MSU crystals in inducing pro-inflammatory monocyte ingress into joints, and the monocyte and macrophage activation central to triggering and mediating gouty joint inflammation [22]. In this context, VSMCs reside in the arterial media in a contractile differentiation state, but VSMC activation and phenotypic switching, including to migrating, proliferating cells, mediates neointima and plaque formation [23]. VSMC proliferation exerts limiting effects in all stages of atherogenesis [23]. By contrast, the senescence and apoptosis of VSMCs, impaired autophagy, and differentiation of VSMCs into macrophage-like cells, and accumulation of cytoplasmic lipid droplets (LDs) and foam cell formation can accelerate atherogenesis and contribute to plaque rupture [24]. Ambient excess of soluble urate is well-recognized to stimulate VSMC proliferation [25]. Yet, to our knowledge, the effects of crystalline MSU deposits on VSMCs have not been studied, despite potential effects on atherogenesis. Thus, we conducted this seminal, exploratory study to characterize how cultured human aortic VSMCs respond to MSU crystals in vitro.

## 2. Materials and Methods

### 2.1. Reagents

All chemicals were from Sigma-Aldrich (St. Louis, MO, USA) unless otherwise indicated. Recombinant platelet-derived growth factor (PDGF)-BB was obtained from R&D System (Minneapolis, MN, USA). Antibodies to phospho-p38 (Thr180/Tyr182) (#4511), phospho-JNK (Thr183/Tyr185) (#4668), LC3B (#3868), and p62 (#8025) were from Cell Signaling Technology, Inc (Danvers, MA, USA). Human oxidized low-density lipoprotein (oxLDL) was purchased from ThermoFisher (#L34357). MSU crystals were prepared under pyrogen-free conditions, using uric acid pretreated for 2 h at 200 °C prior to crystallization [26]. The crystals were suspended at 25 mg/mL in sterile, endotoxin-free phosphate-buffered saline (PBS) and verified to be free of detectable lipopolysaccharide (LPS) contamination (<0.025 endotoxin units/mL) by the Limulus amebocyte lysate assay (BioWhittaker, Inc., Walkersville, MD, USA).

### 2.2. Culture of Human VSMCs

Primary human aortic VSMCs were purchased from ATCC (Gaithersburg, MD) and Lifeline Cell Technology (San Diego, CA, USA), and were maintained in DMEM supplemented with 10% FBS at 37 °C in a 5% CO_2_ humidified atmosphere. For each described experiment with VSMCs, serum-free conditions were used to prevent background effects of factors in whole serum on the responses to stimulation of human VSMCs. VSMCs were not studied beyond the fifth passage.

### 2.3. VSMC Proliferation Assay

VSMCs were plated in each well of a 96-well plate separately, followed by incubation with 10 ng/mL PDGF or MSU crystals (0.1 and 0.2 mg/mL) for 24 h. Cell proliferation rates were detected using the CellTiter 96^®^ AQueous One Solution Cell Proliferation Assay (#G3582) from Promega (Madison, WI, USA).

### 2.4. ELISA Analysis

Human IL-6 and MCP-1 were assayed by ELISA (#DY206 and DY279, from R&D Systems, Minneapolis, MN, USA) via ELISA of conditioned media, by following the manufacturer’s protocols.

### 2.5. Western Blot Analysis

Cells were lysed in RIPA buffer containing protease inhibitor cocktails (Roche, Mannheim, Germany). Cell lysates (10–15 μg) were separated by gradient 4–20% SDS-PAGE, transferred onto Immobilon^®^ PVDF membranes (MilliporeSigma, Burlington, MA, USA), and probed with a primary antibody followed by near-infrared secondary antibodies. Membranes were scanned on a LI-COR Odyssey imaging system (LI-COR Biotech, Lincoln, NB, USA).

### 2.6. Immunostaining

Cells were fixed in 100% cold methanol for 5 min followed by blocking in 1% BSA in PBST (PBS + 0.1% Tween 20) at room temperature for 30 min. After washing with PBS, the cells were incubated with microtubule-associated protein 1A/1B-light chain 3B (LC3B) antibody conjugated with Alexa Fluor 488 and LysoTracker Red, or BODIPY495/503 alone overnight at 40 C. Cells were then washed with PBS, mounted with fluorescence antifade mounting media (#H-1000–10, Vector Lab, Newark, CA, USA), and visualized using a Keyence fluorescence microscope BZ-X710 (Keyence, Itasca, IL, USA).

### 2.7. RNA Sequencing

Cells were collected and subjected to RNA sequencing analysis, performed by LC Sciences (Houston, TX, USA). STRING application—Protein Query was employed to generate a node network of functionally enriched differentially expressed genes (DEGs) whose defined biological processes, cellular content, and molecular functions were analyzed by gene ontology (GO).

### 2.8. Targeted Lipidomics

Methods to assay each class and selected panel of lipid metabolites were previously described in detail [26]. In brief, liquid chromatography–mass spectrometry (LC-MS) analysis of VSMC total sterols was performed. In doing so, human VSMCs were treated with MSU crystals (0.2 mg/mL) or buffer (control) for 24 h. Cells were then subjected to the extraction of a panel of sterols, followed by LC-MS analysis of 19 sterols including 14-demethyl-lanosterol (DML), 25-hydroxycholesterol (OHC), 27-OHC, 7,27-dihydroxycholesterol (dOHC), 7a-OHC, 7a-OH-ONE, 7-DHC, 7-KetoC, 8-DHC, campesterol, cholestanol, cholesterol, desmosterol, dihydrolanosterol (DHL), Dihydro testis meiosis-activating sterol (T-MAS), follicular fluid meiosis-activating sterol (FF-MAS), lanosterol, sitosterol, and zymosterol [26]. The data were normalized to the total protein of cell lysates.

For the gas chromatography–mass spectrometry (GC-MS) analysis [27] of free fatty acids (FFAs), conditioned media of human VSMCs treated with MSU crystals (0.2 mg/mL) or buffer (control) were subjected to extraction of FFAs for 24 h. We assayed a panel of 33 FFAs that included medium-chain FA (MCFA) 12:0, long-chain FA (LCFC) 14:0, 15:0, 16:0, 16:1, 17:0, 17:1, 18:0, 18:1, 18:2, 18:3 N3, 18:3 N6, 18:4, 20:0, 20:1, 20:2, 20:3 N3, 20:3 N6, 20:3 N9, 20:4, 20:5, and very-long-chain FA (VLCFC) 22:0, 22:1, 22:2, 22:3, 22:4, 22:5 N3, 22:5 N6, 22:6, 23:0, 24:0, 24:1, 26:0 [26]. For reversed-phase ultra-performance liquid chromatography–mass spectrometry (RP-UPLC-MS) analysis [27] of eicosanoids, conditioned media were then subjected to extraction followed by analysis of a panel of 54 eicosanoids, including 6k prostaglandin (PG)F1a, PGF2a, PGE2, PGD2, 11b PGF2a, thromboxane (TX)B1, PGF1a, PGE1, d17 6k PGF1a, PGF3a, PGE3, dihomo PGF2a, bicyclo PGE2, 20oh PGF2a, tetranor-PGFM, 12(S)-hydroxy-5Z,8E,10E-heptadecatrienoic acid (12-HHTrE), 11-hydroxyeicosatetraenoic acid (HETE), 11-hydroxyeicosapentaenoic acid (HEPE), 13-hydroxydocoshexaenoic acid (HDoHE), PGA2, PGB2, PGJ2, 15d PGD2, 15d PGJ2, 5-iso PGF2a VI, 8-iso PGF2a III, 8-HDoHE, 16-HDoHE, 20-HDoHE, LTB4, 5-HETE, 5-HEPE, 4-HDoHE, 5,15-diHETE, 7,17-dihydro-dipicolinic acid (dHDPA), 15-HETE, 13-hydroxyoctadecadienoic acid (HODE), 15-hydroxyeicosatrienoic acid (HETrE), 8-HETE, 11-HDoHE, 9-HODE, 9-oxoODE, 18-HETE, 16-HETE, 18-HEPE, 19,20-DiHDPA, 12(13)-EpOME, 8,9-diHETrE, 11,12-diHETrE, 14,15-diHETrE, 9,10-dihydroxyoctadec-12-enoic acid (diHOME), 12,13-diHOME, 20COOH arachidonic acid (AA), and 8-iso-15k PGF2b [26].

### 2.9. Statistical Analyses

GraphPad PRISM 9 (San Diego, CA, USA) was used for statistical analyses. All data were subjected to the normality test. For normally distributed data, either unpaired Student’s *t*-test (comparing 2 groups), one-way (comparing 2 ≥ groups with 1 variable), or two-way analysis of variance (ANOVA) (comparing 2 ≥ groups with 2 independent variables) with Tukey’s multiple comparisons tests were performed. The data are expressed as mean ± SD and *p* < 0.05 was considered statistically significant.

## 3. Results

### 3.1. VSMC IL-6 Release Induced by Human VSMCs

VSMC proliferation was stimulated by the positive control PDGF (10 ng/mL), but not by MSU crystals (Figure 1A). The mitogen-activated protein kinases (MAPKs) transduce the proliferative effects of PDGF in VSMCs [28]. Here, PDGF induced rapid p38 phosphorylation and relatively transient c-Jun N-terminal kinase (JNK) phosphorylation, whereas MSU crystals induced p38 but not JNK phosphorylation (Supplementary Figure S1). PDGF also induced a marked release of the atherogenic cytokines monocyte chemoattractant protein (MCP)-1 and IL-6 (Figure 1B,C). By comparison, MSU crystals robustly induced IL-6 but did not induce MCP-1 (Figure 1B,C).

### 3.2. Marked VSMC Vacuolar Changes Induced by MSU Crystals

MSU crystals, but not PDGF, induced the robust formation of vacuolar structures in VSMCs (Figure 2A and Supplementary Figure S2). To test if the vacuoles represented lipid deposits such as in VSMC macrophage-like foam cell development [29–32], or simply vacuolar changes related to ingested crystals and associated frustrated autophagy [33,34], we treated the cells with bafilomycin A1, a specific vacuolar H + ATPase (V-ATPase) inhibitor. We performed immunostaining with an antibody to LC3B (an autophagosome marker) conjugated with Alexa Fluor 488, and a lysosomal tracker (LysoTracker, Red). As shown in Figure 2B, we thereby documented vacuole formation in VSMCs in response to MSU crystals. In addition, the membranes of most vacuoles stained positively for LC3B (green). Few vacuoles had positive staining for both LC3B and LysoTracker (yellow) in SMCs treated with MSU crystals for 24 h.

Bafilomycin A1 (25 nM) blunted vacuole formation in response to MSU crystals in SMCs. Bafilomycin A1 inhibits autophagy by preventing autophagosome–lysosome fusion [35]. Moreover, not only LC3B-II, the phosphatidylethanolamine (PE)-conjugated form of LC3B, but also p62, which can directly bind to LC3, and is selectively degraded by autophagy, accumulated in VSMCs after 24 h treatment with MSU crystals (Supplementary Figure S2). The results suggested frustrated autophagy flux in a failed autophagic process.

### 3.3. mRNA Changes Induced by MSU Crystals in Human VSMCs

RNA sequencing assayed for differentially expressed genes (DEGs) in human VAMCs in response to MSU crystals. As shown in Figure 3A, MSU crystals upregulated 43 genes (Log2FC ≥ 1) and downregulated 21 genes (Log2FC ≤ −1). STRING enrichment analysis of a network of the 43 upregulated genes indicated one major cluster containing *CHAC1, ATF4, DDIT3, TRIB3,* and *SLC7A5* genes (indicated in red in Figure 3B). Gene ontology (GO) analysis of the network revealed the CHOP–ATF4 complex, cyclic adenosine monophosphate (cAMP) response element-binding protein (CREB), and the intrinsic apoptosis signaling pathway in response to endoplasmic reticulum (ER) stress as the major enriched signaling pathways in the GO terms of cellular component, molecular function, and biological process, respectively (Figure 3C). MSU-crystal-induced changes in mRNA levels of two members of the nuclear receptors of the subfamily 4 group A1 and A3 (NR4A1 and NR4A3), known to be induced by atherogenic stimuli in VSMCs [36], were shown in another cluster (*NR4A1, NR4A3*, *FOSB*, and *DUSP*, indicated in yellow in Figure 3B).

GABARAPL1 (GABAA-receptor-associated protein-like 1), which suppresses autophagic flux, was downregulated (Log2FC= −1.7) in response to MSU crystals. These results potentially reflected a compensatory change to the observed impairment of autophagy flux in MSU-crystal-treated VSMCs (Supplementary Figure S3). Last, we observed that MSU crystals induced downregulation of the VSMC cytoskeletal and differentiation and function regulators LMOD1 (leiomodin 1) and SYNPO2 (synaptopodin 2).

### 3.4. Effects of MSU Crystals on Intracellular Cholesterol Metabolism in Human VSMCs

In the cytosol of VSMCs treated with MSU crystals for 24 h, we observed increased staining by BODIPY495/503, a lipophilic fluorescent probe that labels cellular neutral lipid contents, particularly so for those localized to lipid droplets (Supplementary Figure S4). MSU-crystal-induced formation of vacuoles under these conditions was not associated with detectable staining of BODIPY inside the vacuoles. Targeted lipidomics examined the effects of MSU crystals on cellular lipid metabolism in VSMCs. Since MSU crystals avidly bind LDL, with effects on phagocyte activation [37], these studies were performed in the presence of atherogenic oxLDL [38].

Whether acquired from extracellular lipoproteins or synthesized de novo, cholesterol undergoes metabolic conversion into not only bile acids and steroid hormones but also oxidized sterols (oxysterols) [38]. Such oxysterols are present in human atherosclerotic plaques and are strongly atherogenic [38]. Here, LC-MS analysis of a panel of 19 sterols (Figure 4A) revealed that MSU crystals alone induced intracellular increases in the cholesterol biosynthetic intermediates 14-DML, desmosterol [39], and zymosterol (Figure 4B–D). Several intracellular oxysterols, such as 25-OHC, 27-OHC, 7,27-dOHC, 7alpha-OHC, and 7-ketocholesterol (7-KC), were significantly elevated by oxLDL alone (Figure 4E–I). MSU-crystal-induced elevation of 25-OHC and 27-OHC levels was not statistically significant. By contrast, combined MSU crystal and oxLDL treatment significantly increased 27-OHC.

### 3.5. Impact of MSU Crystals on VSMC Intracellular Free Fatty Acids (FFAs)

We analyzed a panel of 33 FFAs, including medium-chain fatty acids (MCFAs) and long-chain fatty acids (LCFAs) (Figure 5A), by gas chromatography–mass spectrometry (GC-MS) in VSMCs treated with MSU crystals in the presence or absence of oxLDL. MSU crystal treatment alone had little effect on the levels of the analyzed FFAs, whereas oxLDL significantly increased the levels of two unsaturated FFAs, linoleic acid (18:2) and erucic acid (22:1). When compared to oxLDL alone, the combination of MSU crystals and oxLDL induced significant reduction in multiple FAs, including linoleic acid (18:2), palmitoleic acid (16:1), oleic acid (18:1), arachidonic acid (20:4), erucic acid (22:1), and docosahexaenoic acid (DHA 22:6) (Figure 5B,C).

### 3.6. Effects of MSU Crystals on VSMC Eicosanoid Release

We used RP-UPLC-MS to analyze a panel of 80 eicosanoids from VSMCs treated with MSU crystals (Figure 6A) in conditioned media. Where indicated, oxLDL was present. Of the 15 eicosanoids affected by treatment with MSU crystals or oxLDL, approximately two-thirds were derived from AA via enzymatic (e.g., cyclooxygenases (COXs) and lipoxygenases (LOXs)) and non-enzymatic oxidation (Figure 6B). Certain AA-derived, eicosapentaenoic acid (EPA)-derived, docosahexaenoic acid (DHA)-derived, and linolenic acid (LA)-derived metabolites were increased only by oxLDL. Notably, levels of AA were not altered by MSU crystals or oxLDL alone. By contrast, the marked induction of AA release by the combination of MSU crystals and oxLDL suggested a synergistic effect. The AA derivative 6-keto-Prostaglandin F1a (6K PGF1a) was increased significantly by MSU crystals alone, and this was enhanced when oxLDL was also present. Last, levels of prostaglandin E2 (PGE2) and 13,14-Dihydro-15-keto-PGF2a (dhk PGF2a) were increased by the combination of MSU crystals and oxLDL.

## 4. Discussion

MSU crystals have been detected within coronary atherosclerotic plaques, potentially a consequence of enhanced lesion XO and urate content. This seminal study revealed the potential of MSU crystals to activate VSMCs in vitro, with effects that can enhance atherogenesis. A prime example was the observation that MSU crystals induced IL-6 release by VSMCs. IL-6 atherogenic effects include the promotion of inflammation and foam cell formation, and stimulation of altered VSMC differentiation, including ectopic osteoblastic differentiation in the artery wall and associated arterial calcification [40,41].

In this study, VSMC RNA sequencing and GO analysis demonstrated that MSU crystals upregulated the mRNA of the CHOP–ATF4 complex, CREB, and the intrinsic apoptosis signaling pathway in response to ER stress as the major cellular component, molecular function, and biological process, respectively. In this light, the ER stress response modulates atherogenesis and is particularly upregulated in advanced atherosclerotic plaques [42]. Despite the beneficial effect of a transient unfolded protein response (UPR), prolonged ER-stress-induced CHOP expression is involved in VSMC apoptosis both in vitro and in vivo, and it promotes thinning of the protective collagen cap of the plaque, and consequent plaque vulnerability [23,24,42–44]. The ER stress effector ATF4 plays a critical role in the pathogenesis of arterial lesion calcification through increased phosphate uptake in VSMCs [45]. The nuclear transcription factor CREB, which is downregulated in vascular disease, is a central modulator of cell proliferation, differentiation, adaptation, and survival under stress [46].

A particularly striking VSMC response to MSU crystals was the robust development of vacuoles, associated with evidence for impaired autophagic flux. RNA sequencing demonstrated that the VSMC mRNA of GABARAPL1, which suppresses autophagic flux [47], was downregulated in response to MSU crystals. This result suggested an adaptive response to impaired autophagy. In prior studies, MSU crystals induced autophagy of osteoblasts and chondrocyte autophagy [48,49], which, like VSMCs, are not professional phagocytes. In VSMCs, autophagy is not simply induced by metabolic stress but also by diverse stimuli, such as oxidative stress and oxidized lipids, as well as a number of cytokines and growth factors [23,24]. VSMC autophagy is intimately involved in promoting survival as the cells undergo the ER stress response and phenotypic switching [23,24].

Additional RNA sequencing findings in this study buttressed the potential atherogenic effects of MSU crystals. First, we observed that MSU crystals decreased the mRNA of SYNPO2 and of LMOD1, a downstream target of the VSMC master regulator serum response factor (SRF)–Myocardin (MYOCD) complex [50]. Both LMOD1 and SYNPO2 play major roles in the maintenance of smooth muscle actin polymerization and contractile differentiation, and reduced LMOD1 and SYNPO2 is linked with altered VSMC phenotype in arterial disease in vivo [50,51]. Second, NR4A1 and NR4A3 mRNA were upregulated in response to MSU crystals. These NR4A receptors are increased in VSMCs by atherogenic stimuli and upregulated in human atherosclerotic plaque neointima [36].

Lipidomics demonstrated that MSU crystals alone increased the levels of the cholesterol biosynthetic intermediates 14-DML, desmosterol, and zymosterol (Figure 4B–D). Oxysterols are associated with nearly every atherogenesis pathway, are present in human atherosclerotic plaques, and are held to play an active role in plaque development [38]. Conversely, since desmosterol inhibits NLRP3 inflammasome and macrophage activation and limits atherosclerosis [39], not all the MSU-crystal-induced changes in the VSMC sterol panel were atherogenic.

LDL binds avidly to MSU crystals and markedly inhibits MSU-crystal-induced activation of neutrophils [37]. However, LDL undergoes oxidation in atherosclerotic lesions, is taken up robustly by VSMCs and macrophages, and is atherogenic [38]. Here, the combination of MSU crystals and oxLDL significantly increased the VSMC levels of 27-OHC, the major oxysterol in advanced atherosclerotic lesions [52]. This suggested the possibility, which warrants testing, that OxLDL bound to MSU crystals may undergo increased VSMC uptake and further oxidation. Elevation of 27-OHC promotes murine experimental atherosclerotic lesion formation without altered lipid status [52]. Furthermore, 27-OHC attenuates estrogen-related atheroprotection and elevates vascular inflammation via estrogen receptor alpha [52].

Lipidomics revealed that MSU crystals alone had little effect on intracellular FFAs. However, MSU crystals and oxidized LDL synergistically induced a marked release of arachidonate, while decreasing intracellular linoleic acid (18:2), palmitoleic acid (16:1), oleic acid (18:1), arachidonic acid (20:4), erucic acid (22:1), and docosahexaenoic acid (DHA 22:6). We did not determine if the decrease in the levels of particular intracellular FFAs was due to increased degradation or reduced synthesis of FFAs. The release of 6K PGF1a was increased significantly by MSU crystals alone. Levels of released PGE2, which is a central mediator of the inflammatory response, and dhk PGF2a, a metabolite of PGF2a that increases following tissue injury, were also increased by the combination of MSU crystals and oxLDL.

The ω−6 PUFA arachidonic acid is a substrate for the biosynthesis of PGs and leukotrienes (LTs) that mediate inflammation, including in the vasculature [53]. That said, arachidonic acid can also be used to biosynthesize lipoxin A4 (LXA4), which participates in the resolution of inflammation. Arachidonic acid metabolites modulate vascular tone and contribute to cardiovascular diseases, including hypertension, atherosclerosis, and myocardial infarction [53]. The polyunsaturated ω−6 FA linoleic acid can be converted to longer-chain anti-inflammatory ω−3 FAs, such as EPA and DHA, or to longer-chain ω−6 FAs, such as AA [54].

The limitations of this seminal study are principally related to its exploratory and in vitro nature, the focus on the effects of MSU crystals, and the requisite contraction of the experimental template to render it manageable. In this context, though MSU crystals are reported in atherosclerotic plaques, it was beyond the scope of the work to compare the effects of the other crystal types and cells found in plaques or to assess combinations of MSU with cholesterol or BCP crystals. MSU crystals can prime the subsequent enhancement of innate immune tissue inflammatory reactions through effects on macrophages and fibroblasts [22]. Thus, it would also be of interest to directly assess in vivo if MSU crystals can enhance atherogenesis and plaque instability, and if MSU crystals share or diverge from the effects of crystals of cholesterol and BCP on these processes. Importantly, complex, extensive distinctions exist in atherogenesis-regulating effects between individual eicosanoids, reviewed in great detail elsewhere [55–58], that could ultimately be relevant to the seminal findings of this study. Last, our experimental template did not test for MSU crystal effects on all potentially atherogenic VSMC cytokine release responses, calcification, and changes in cell differentiation, such as senescence.

## 5. Conclusions

We conclude that MSU crystals induced severe VSMC vacuole formation and altered cell homeostasis mechanisms, including frustrated autophagy, that were associated with altered transcriptional pathways, IL-6 release, intracellular accumulation of atherogenic oxysterols, and eicosanoid release.

## Supplementary Material

Supplementary Figures

**Supplementary Materials:** The following supporting information can be downloaded at: https://www.mdpi.com/article/10.3390/gucdd1030016/s1, Figure S1: Rapid induction of phosphorylation of p38 but not JNK by MSU crystals in human SMCs; Figure S2: MSU crystals but not PDGF robustly induced vacuole formation in human VSMCs; Figure S3: Time-dependent accumulation of LC3B and p62 by MSU crystals in human VSMCs; Figure S4: Neutral lipid contents in the cytoplasm of human VSMCs increased by MSU crystals.

## Figures and Tables

**Figure 1.**
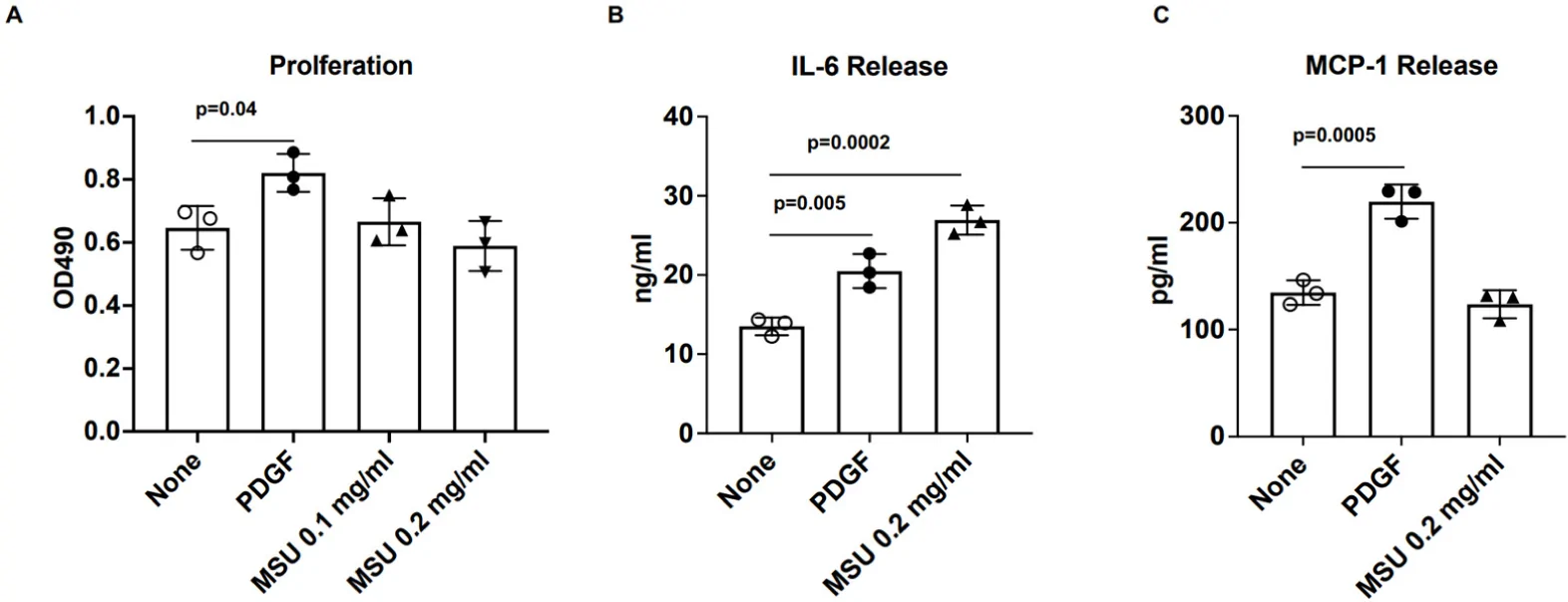
Differential effects of MSU crystals and PDGF on proliferation and cytokine production in human VSMCs. Three different donors of human VSMCs (passage number <3–4) were stimulated with MSU crystals (0.1 and 0.2 mg/mL) and PDGF (10 ng/mL) for 24 h under serum-free conditions. Cell proliferation was evaluated (**A**) using the CellTiter 96^®^ AQueous One Solution Cell Proliferation Assay (Promega) and production of MCP-1 (**B**) and IL-6 (**C**) was measured by ELISA analysis of the conditioned media. One-way ANOVA with Tukey’s post hoc comparison test was used for data analysis.

**Figure 2.**
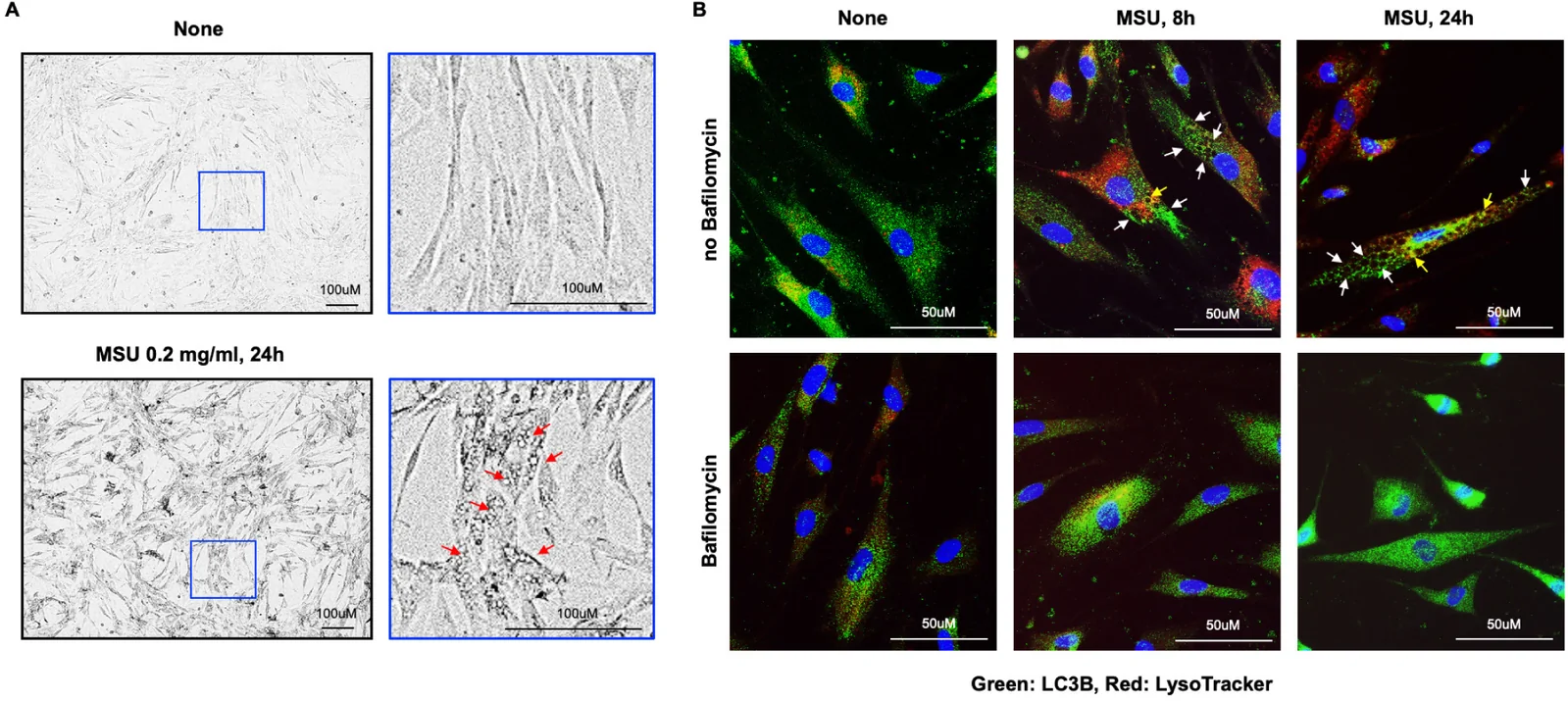
Marked autophagic vacuole formation induced by MSU crystals in human VSMCs, which was inhibited by Bafilomycin. Human VSMCs were stimulated with MSU crystals (0.2 mg/mL) for 8 and 24 h in the presence or absence of vacuolar H+ ATPase (V-ATPase) inhibitor Bafilomycin (50 nM). Many vacuoles were formed in the cytoplasm of VSMCs as seen in the images of phase contrast, pointed out by red arrows (**A**). Immunostaining with LC3B antibody conjugated with Alexa Fluor 488 and LysoTracker Red showed that the membranes of many vacuoles were LC3B-positive (white arrows) but few of them were positive for both LC3B and LysoTracker Red (yellow arrows) in the top panel (**B**). No vacuoles were observed when Bafilomycin was present (bottom panel, (**B**). The images shown are representative of studies performed using cells from 3 different biological donors.

**Figure 3.**
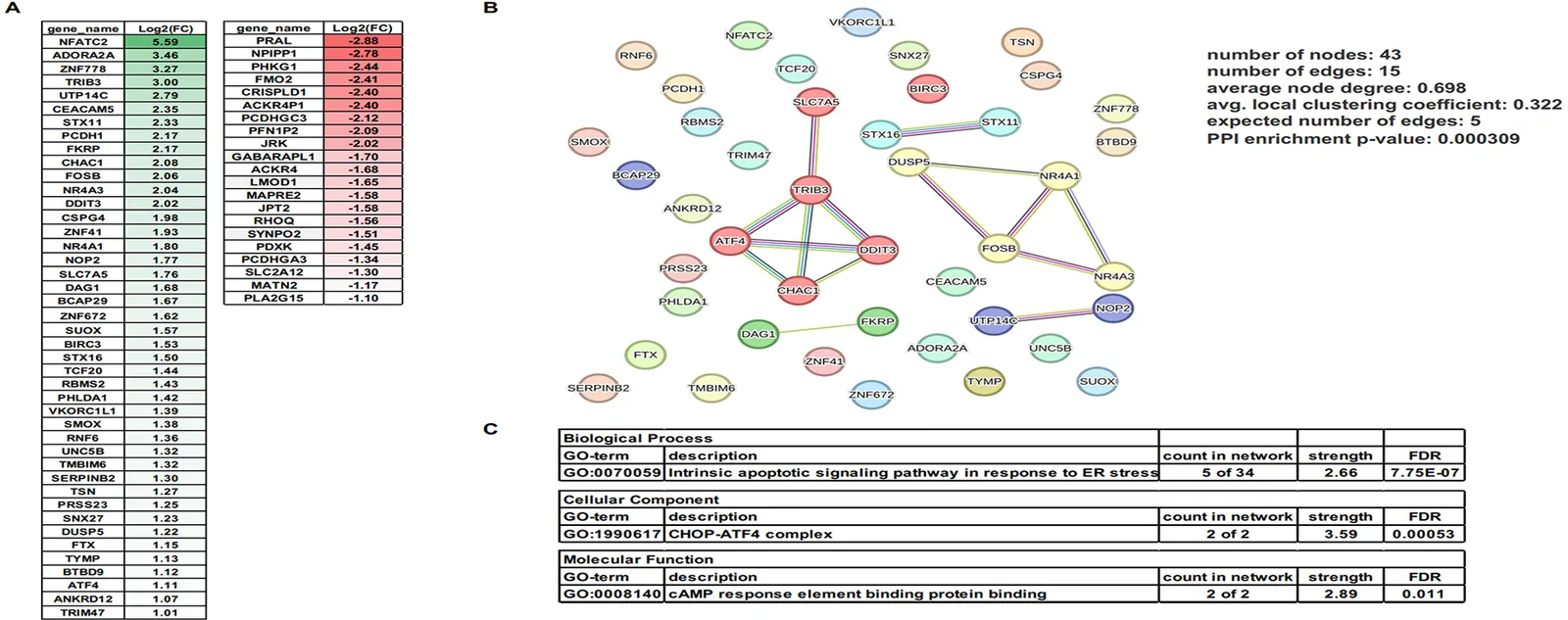
RNA-seq analysis gene expression changes induced by MSU crystals in human VSMCs. Three different donors of human VSMCs (passage number <3–4) were stimulated with MSU crystals (0.2 mg/mL) for 6 h. Total RNA was extracted and subjected to transcriptomics analysis by RNA-seq. The mean values of DEGs either upregulated (log2FC ≥ 1) or downregulated (Log2FC ≤ −1) from 3 different donors are listed in the table (**A**). STRING enrichment analysis of 43 upregulated genes is shown in (**B**). Gene ontology analysis of the biological process, cellular component, and molecular function are shown in (**C**).

**Figure 4.**
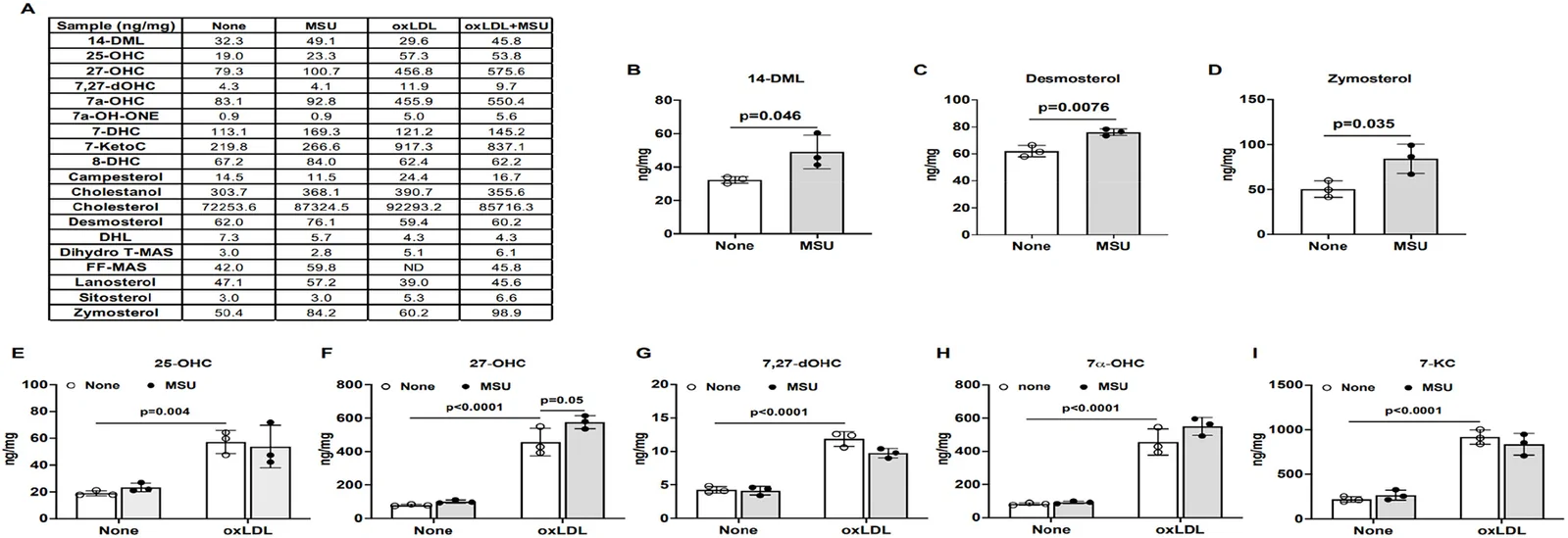
Effects of MSU crystals and oxLDL on cholesterol metabolism in human VSMCs. Three different donors of human VSMCs (passage number <3–4) were stimulated with MSU crystals (0.2 mg/mL), oxLDL (40 mg/mL), and a combination of MSU crystals with oxLDL at the same concentrations for 24 h. The intracellular cholesterol metabolism was evaluated by LC-MS analysis of a panel of 19 sterols. The mean values of the sterols are shown in the table (**A**). Three sterols, including 14-demethyl-lanosterol (14-DML), desmosterol, and zymosterol, were significantly induced by MSU crystals (**B**–**D**). Oxysterols such as 25-hydroxycholesterol (25-OHC), 27-OHC, 7,27-dihydroxycholesterol (dOHC), 7alpha-OHC, and 7-ketocholesterol (7-KC) were only significantly elevated by oxLDL not by MSU crystals except 27-OHC (**E**–**I**). Two-way ANOVA with Tukey’s post hoc comparison test was used for data analysis.

**Figure 5.**
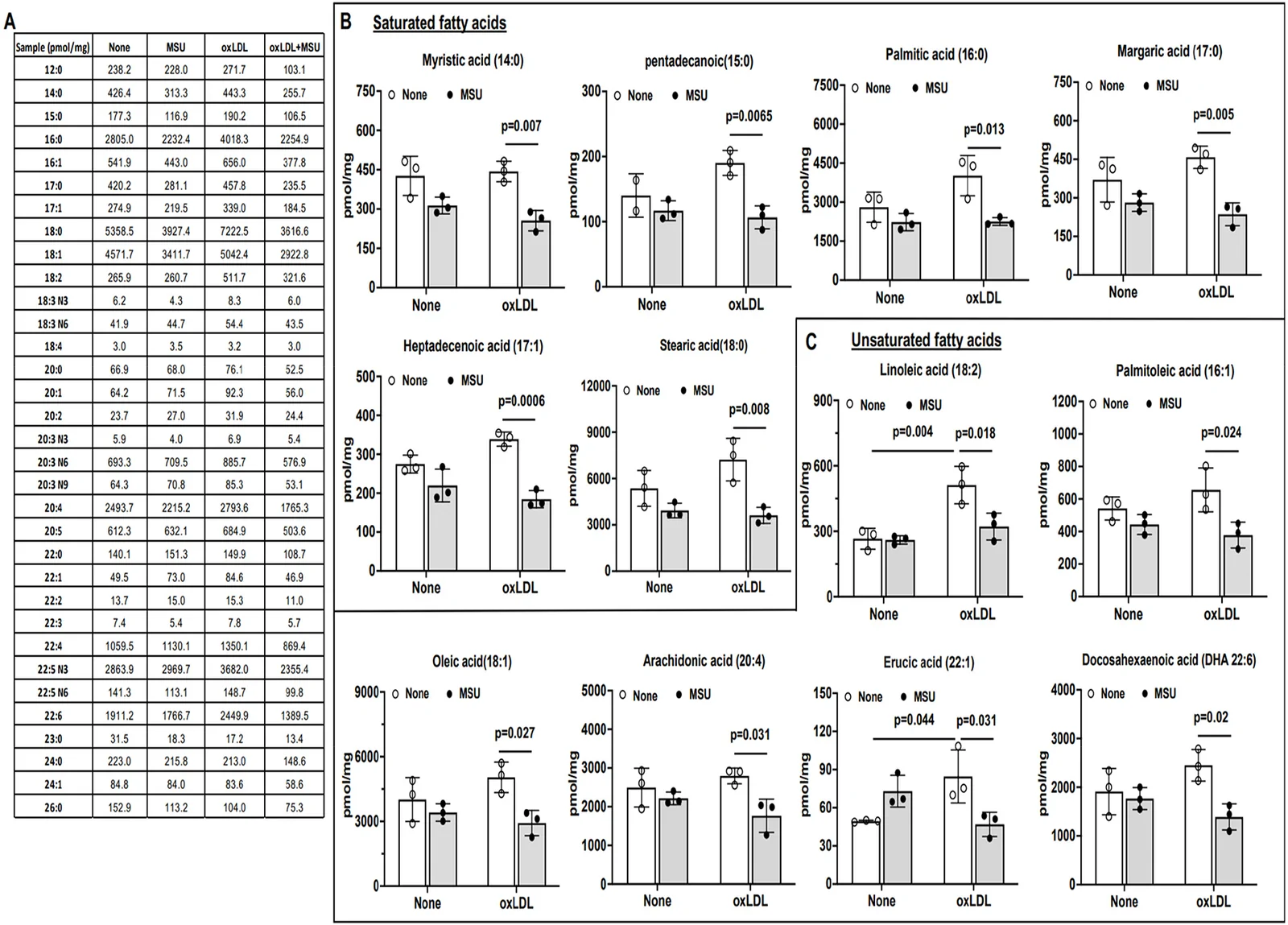
Effects of MSU crystals and oxLDL on intracellular free fatty acids (FFAs) in human VSMCs. Three different donors of human VSMCs (passage number < 3–4) were stimulated with MSU crystals (0.2 mg/mL), oxLDL (40 mg/mL), and a combination of MSU crystals with oxLDL at the same concentrations for 24 h. The intracellular FFAs were evaluated by GC-MS analysis of 33 FFAs (**A**). The mean values of FFAs are shown in the table (**A**). MSU crystals alone showed little impact on FFAs. OxLDL alone caused an increase in 2 unsaturated FFAs, linoleic acid (18:2) and erucic acid (22:1) (**C**). When MSU crystals were combined with oxLDL, several saturated and unsaturated FFAs were significantly reduced (**B**,**C**). Two-way ANOVA with Tukey’s post hoc comparison test was used for data analysis.

**Figure 6.**
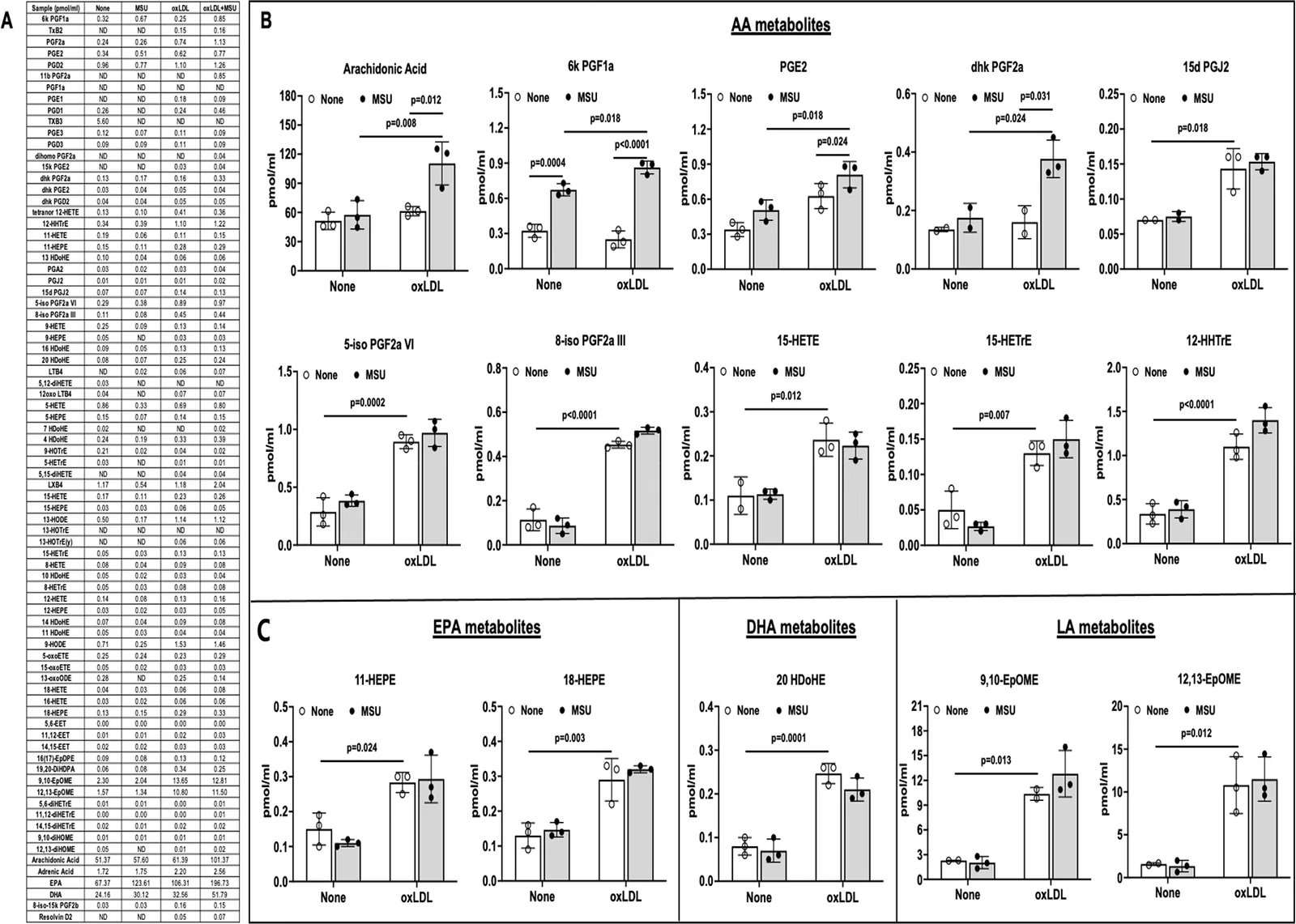
Effects of MSU crystals and oxLDL on eicosanoid release from human VSMCs. Three different donors of human VSMCs (passage number <3–4) were stimulated with MSU crystals (0.2 mg/mL), oxLDL (40 mg/mL), and a combination of MSU crystals with oxLDL at the same concentrations for 24 h. The conditioned media were subjected to RP-UPLC-MS for the analysis of 80 eicosanoids (**A**). The mean values of eicosanoids are shown in the table (**A**). The levels of several metabolites derived from AA that were affected by MSU crystals alone, oxLDL alone, or a combination of both are shown in (**B**). The levels of some metabolites of EPA, DHA, and LA that were increased only by oxLDL are shown in (**C**). Two-way ANOVA with Tukey’s post hoc comparison test was used for data analysis.

## Data Availability

Data supporting reported results can be requested from R.T. and R.L.-B. and RNA sequencing date will be deposited in the GEO database.

## Abbreviations

MSU: monosodium urate
VSMCs: vascular smooth muscle cells
DEGs: differentially expressed genes
ER: endoplasmic reticulum
AA: arachidonic acid
XO: xanthine oxidase
CNS: central nervous system
BCP: basic calcium phosphate
DECT: dual-energy computed tomography
LDs: lipid droplets
PDGF: platelet-derived growth factor
oxLDL: oxidized low-density lipoprotein
PBS: phosphate-buffered saline
LPS: lipopolysaccharide
LC3B: microtubule-associated protein 1A/1B-light chain 3B
LC-MS: liquid chromatography–mass spectrometry
FFAs: free fatty acids
GC-MS: gas chromatography–mass spectrometry
MCFA: medium-chain free fatty acid
LCFC: long-chain free fatty acid
VLCFC: very-long-chain fatty acid
RP-UPLC-MS: reversed-phase ultra-performance liquid chromatography–mass spectrometry
ANOVA: analysis of variance
MAPKs: mitogen-activated protein kinases
JNKs: c-Jun N-terminal kinases
MCP: monocyte chemoattractant protein
cAMP: cyclic adenosine monophosphate
CREB: cAMP response element-binding protein
COX: cyclooxygenase
LOX: lipoxygenase
PG: prostaglandin
TX: thromboxane
12-HHTrE: 12(S)-hydroxy-5Z,8E,10E-heptadecatrienoic acid
HETE: hydroxyeicosatetraenoic acid
HEPE: hydroxyeicosapentaenoic acid
HDoHE: hydroxydocoshexaenoic acid
dHDPA: dihydro-dipicolinic acid
HODE: hydroxyoctadecadienoic acid
HETrE: hydroxyeicosatrienoic acid
diHOME: dihydroxyoctadec-12-enoic acid
EPA: eicosapentaenoic acid
DHA: docosahexaenoic acid
AA: arachidonic acid
LA: linolenic acid
14-DML: 14-demethyl-lanosterol
OHC: hydroxycholesterol
DHC: dihydroxycholesterol
DHL: dihydrolanosterol
T-MAS: testis meiosis-activating sterol
FF-MAS: follicular fluid meiosis-activating sterol
dOHC: dihydroxycholesterol
KC: ketocholesterol
GABARAPL1: GABA type A receptor-associated protein-like 1
LMOD1: leiomodin 1
SYNPO2: synaptopodin 2
ATF4: activating transcription factor 4
CHOP (DDIT3): C/EBP homologous protein (DNA-damage-inducible transcript 3)
NR4A1: nuclear receptor subfamily 4 group A member 1
NR4A3: nuclear receptor subfamily 4 group A member 3
FOSB: FosB proto-oncogene, AP-1 transcription factor subunit
DUSP: dual-specificity phosphatase
CHAC1: ChaC glutathione-specific gamma-glutamylcyclotransferase 1
TRIB3: tribbles pseudokinase 3
SLC7A5: solute carrier family 7 member 5
GABARAPL1: GABAA-receptor-associated protein-like 1
MYOCD: myocardin
SRF: serum response factor
UPR: unfolded protein response
LT: leukotriene
LXA4: lipoxin A4
