# Supplementary material for: Atherogenic Activation of Human Vascular Smooth Muscle Cells by Monosodium Urate Crystals
Source: Gout Urate Cryst Depos Dis. Author manuscript; Available in PMC 2026 Oct 17. (PMC13673954; doi:10.3390/gucdd1030016)
Supplement: Supplementary file 1 [file NIHMS2203241-supplement-Supplementary_Figures.pdf]

Supplemental Figure 1. Rapid induction of phosphorylation of p38 but not JNK by MSU crystals in human SMCs

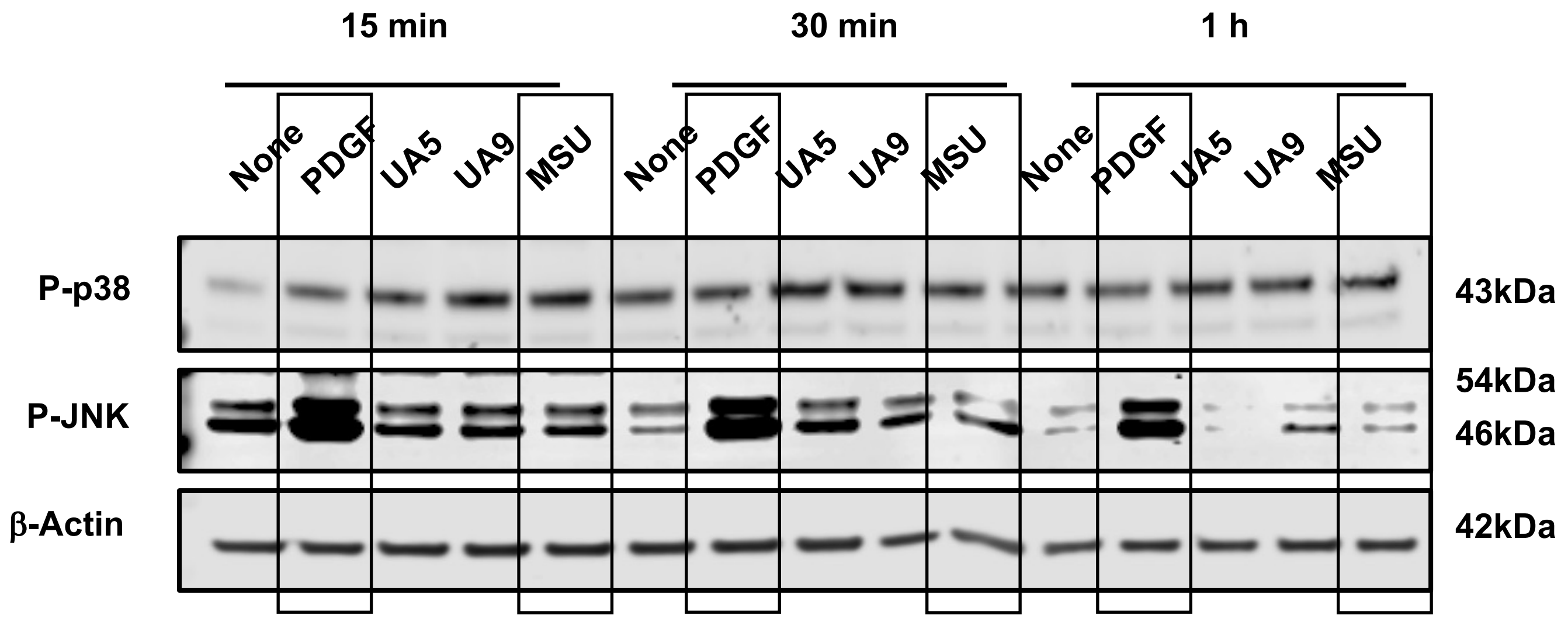

Supplemental Figure 1. Rapid induction of phosphorylation of p38 but not JNK by MSU crystals in human VSMCs. Human VSMCs were stimulated with PDGF (10 ng/ml), soluble uric acid at 5 and 9 mg/dL, or MSU crystals (0.2 mg/ml) for 15, 30 and 60 minutes. The cell lysates were subjected to Western blot analysis of phospho-p38 (Thr180/Tyr182) and phospho-JNK (Thr183/Tyr185). Compared to PDGF, MSU crystals only rapidly induced phosphorylation of p38 but not JNK. Data shown were representative of studies performed using cells from 3 different biological donors.

Supplemental Figure 2. MSU crystals but not PDGF robustly induced vacuole formation in human VSMCs

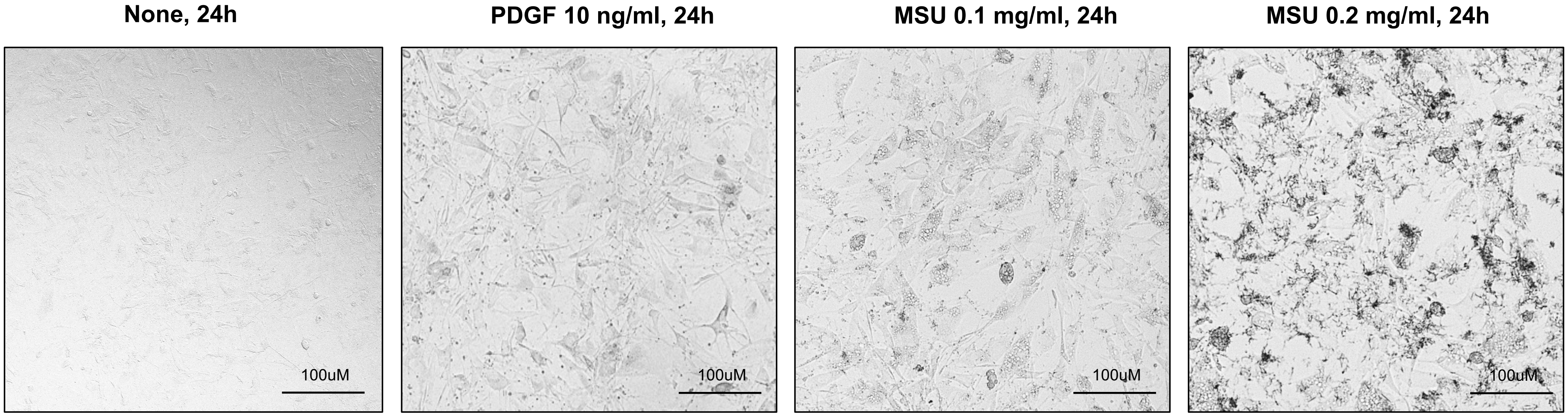

Supplemental Figure 2. MSU crystals but not PDGF robustly induced vacuole formation in human VSMCs. Human VSMCs were stimulated with PDGF (10 ng/ml) or MSU crystals (0.1 and 0.2 mg/ml) for 24 hours. Formation of vacuoles were only observed by MSU crystals but not PDGF. Data shown were representative of studies performed using cells from 3 different biological donors.

Supplemental Figure 3. Time-dependent accumulation of LC3B and p62 by MSU crystals in human VSMCs.

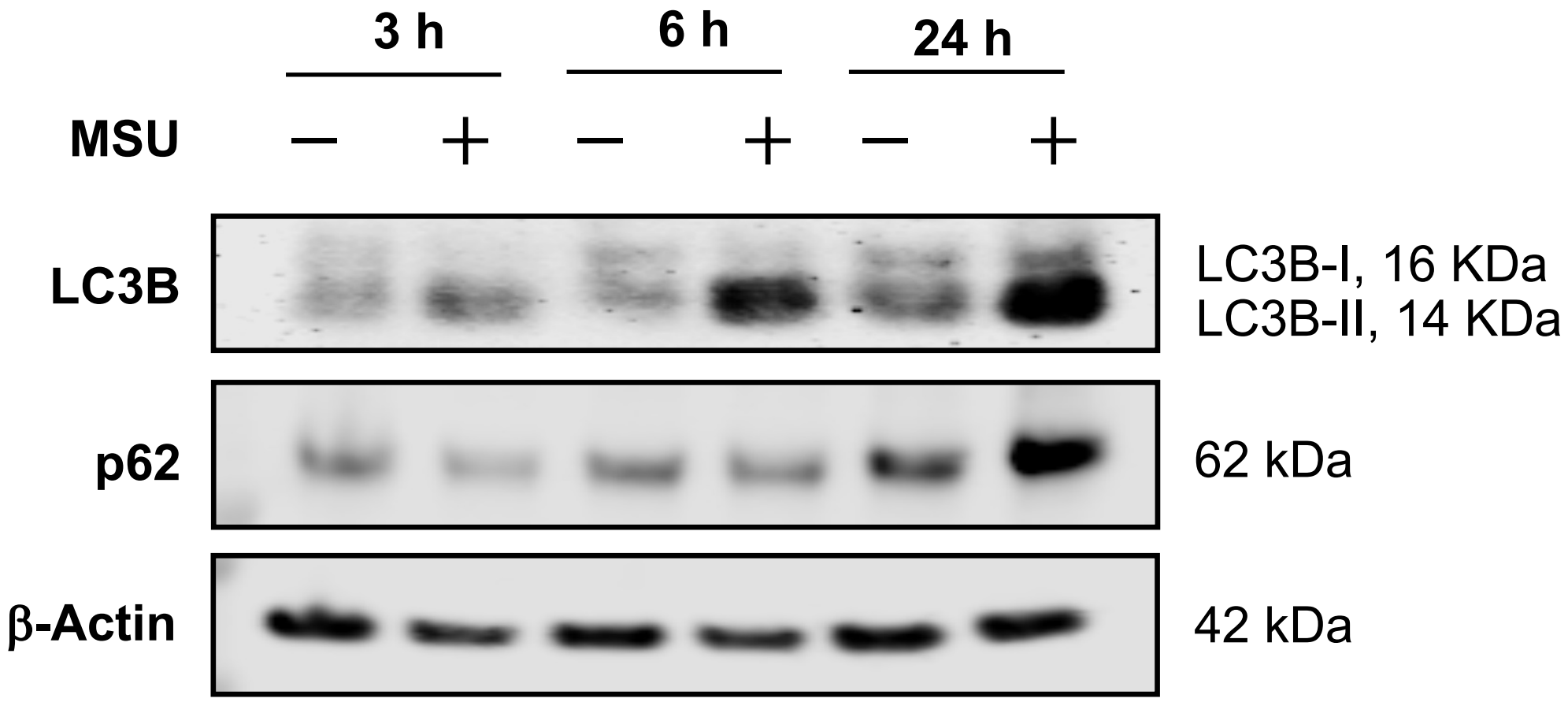

Supplemental Figure 3. Time-dependent expression of LC3B and p62 by MSU crystals in human VSMCs. Human VSMCs were stimulated with MSU crystals (0.2 mg/ml) for 3, 6 and 24 hours. The cell lysates were subjected to Western blot analysis of expression of LC3B and p62. Expression of LC3B-II and p62 were accumulated in VSMCs after 24 h treatment with MSU crystals, indicating impaired autophagy flux. Data shown were representative of studies performed using cells from 3 different biological donors.

Supplemental Figure 4. Increased neutral lipid contents in cytoplasm of human VSMCs by MSU crystals.

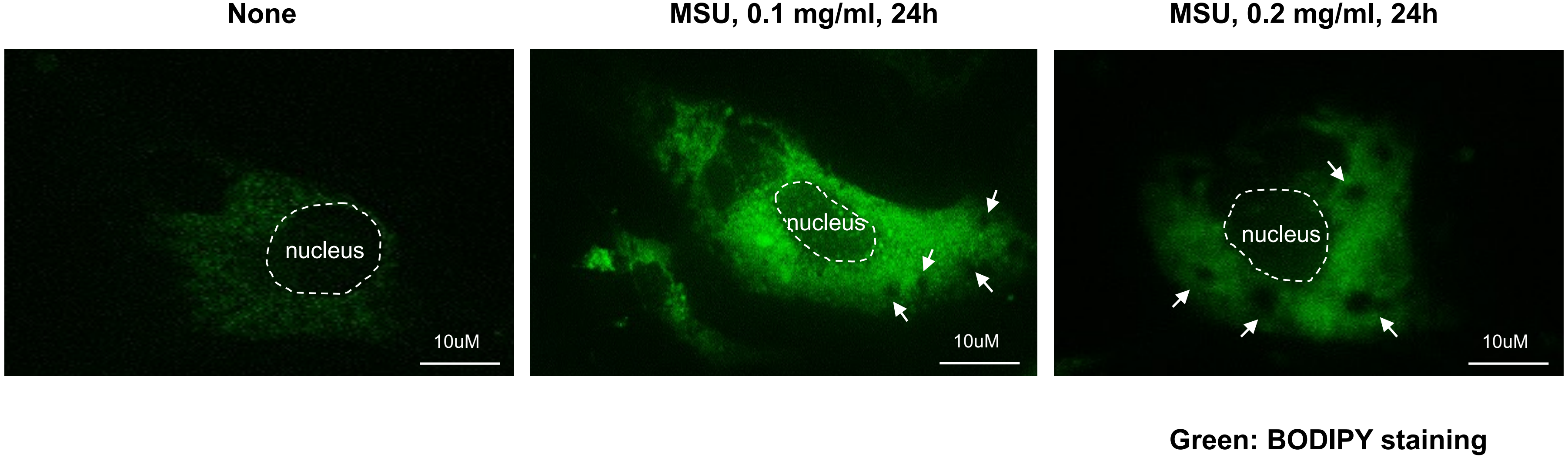

Supplemental Figure 4. Increased neutral lipid content in cytoplasm of human VSMCs by MSU crystals. Human VSMCs were stimulated with MSU crystals (0.1 and 0.2 mg/ml) for 24 hours. The cells were then stained with BODIPY495/503, a lipophilic fluorescent probe used to label cellular neutral lipid contents. Increased intensity of BODIPY staining (green) in the cytosol of VSMCs treated with MSU crystals were observed. No BODIPY staining was seen inside the vacuoles (pointed by white arrows). Data shown were representative of studies performed using cells from 3 different biological donors.
